# Exopolysaccharide-Independent Social Motility of *Myxococcus xanthus*

**DOI:** 10.1371/journal.pone.0016102

**Published:** 2011-01-05

**Authors:** Wei Hu, Muhaiminu Hossain, Renate Lux, Jing Wang, Zhe Yang, Yuezhong Li, Wenyuan Shi

**Affiliations:** 1 School of Dentistry, University of California Los Angeles, Los Angeles, California, United States of America; 2 Molecular Biology Institute, University of California Los Angeles, Los Angeles, California, United States of America; 3 State Key Laboratory of Microbial Technology, School of Life Science, Shandong University, Jinan, China; Newcastle University, United Kingdom

## Abstract

Social motility (S motility), the coordinated movement of large cell groups on agar surfaces, of *Myxococcus xanthus* requires type IV pili (TFP) and exopolysaccharides (EPS). Previous models proposed that this behavior, which only occurred within cell groups, requires cycles of TFP extension and retraction triggered by the close interaction of TFP with EPS. However, the curious observation that *M. xanthus* can perform TFP-dependent motility at a single-cell level when placed onto polystyrene surfaces in a highly viscous medium containing 1% methylcellulose indicated that “S motility” is not limited to group movements. In an apparent further challenge of the previous findings for S motility, mutants defective in EPS production were found to perform TFP-dependent motility on polystyrene surface in methylcellulose-containing medium. By exploring the interactions between pilin and surface materials, we found that the binding of TFP onto polystyrene surfaces eliminated the requirement for EPS in EPS^-^ cells and thus enabled TFP-dependent motility on a single cell level. However, the presence of a general anchoring surface in a viscous environment could not substitute for the role of cell surface EPS in group movement. Furthermore, EPS was found to serve as a self-produced anchoring substrate that can be shed onto surfaces to enable cells to conduct TFP-dependent motility regardless of surface properties. These results suggested that in certain environments, such as in methylcellulose solution, the cells could bypass the need for EPS to anchor their TPF and conduct single-cell S motility to promote exploratory movement of colonies over new specific surfaces.

## Introduction

*Myxococcus xanthus* is a Gram-negative soil bacterium with a complex life cycle including diverse social behaviors such as “group hunting” and fruiting body formation [1], [2]. A crucial feature of *M. xanthus* behavior is its ability to move in the direction of cell's long axis on solid surfaces by a flagella-independent mechanism called "gliding" [3]. Gliding motility is widespread in nature and has been shown to be essential in biofilm formation and the pathogenesis of motile species [4], [5], [6], [7], [8]. Genetic and phenotypic analyses have shown that *M. xanthus* gliding motility is regulated by the A (adventurous) and the S (social) motility systems [9], [10]. The A motility system allows movement of isolated cells and does not require cell-cell contact, while the S motility system is typically employed for coordinated group movement of cells. The two systems appear to operate independently [11] but in a coordinated fashion [12], and it has been suggested that each motility system provides different selective advantages to cells on surfaces containing different concentrations of agar, while enabling *M. xanthus* to adapt to a variety of physiological and ecological environments [13].

S motility in *M. xanthus* is mechanistically equivalent to twitching motility, the flagella-independent form of bacterial translocation over moist surfaces employed by *Pseudomonas aeruginosa* and *Neisseria gonorrhoeae* [7]. Mutations that abolish S motility (for a recent review, see [3]) normally affect type IV pili (TFP) biogenesis [14], the exopolysaccharide (EPS) component of the extracellular matrix (ECM) [15], [16], [17] or the lipopolysaccharide O-antigen [18], [19], [20]. Further functional studies have shown that S motility is powered by cycles of TFP extension and retraction [21], [22] and depend on the interaction of TFP with EPS [23].

The highly organized and coordinated process of movement with cell-cell contact in a community of cells appears to be the normal state of affairs in S motility [9], [23], [24], [25], [26] as well as twitching motility [7], [27]. However, it has been demonstrated that individual isolated cells can show TFP-dependent movement in *M. xanthus* [22], *P. aeruginosa* [28] and *N. gonorrhoeae* [29] when in contact with certain substrates (for a review, see [7]). *M. xanthus* strains deficient in A motility are capable of moving as single cells on a polystyrene surface in 1% methylcellulose [22], even though on 1.5% agar gliding of individual cells requires A motility [9], [10]. It is not fully understood how the methylcellulose-containing system compensates for the absence of the cell-cell contact normally necessary for S motility in *M. xanthus* [30]. *M. xanthus* cells have been observed to conduct single-cell S motility only on some surfaces with high water content [22], while the biological relevance of this unique behavior has not been established. In this study, we conducted a detailed investigation of S motility of *M. xanthus* and a variety of mutant derivatives in methylcellulose-containing medium to analyze these apparent discrepancies, which led to further information about the roles of TFP and EPS in S motility.

## Results

### *M. xanthus* cells lacking EPS displayed TFP-dependent motility on polystyrene surfaces in methylcellulose

In *M. xanthus,* A motility is advantageous on relatively firm and dry surfaces (e.g. 1.5% agar), while S motility is dominant on wet surfaces (e.g. on 0.3% agar or in 1% methylcellulose) [13], [22]. *M. xanthus* and different mutants derivatives lacking distinct motility features were systematically compared regarding their motility in methylcellulose relative to their S motility on 0.3% agar surface (Table 1). Consistent with previous findings [13], A^−^S^+^ cells, like strain MXH1216 (A::*Tn5*) and MXH2265 (*ΔaglZ*), showed S motility mainly in cell groups, while A^−^S^−^ cells (including EPS^−^ and pilus^−^ cells) were not motile by S motility on 0.3% agar. In contrast, on polystyrene surfaces in 1% methylcellulose, S motility was frequently detected in both isolated and grouped S^+^ *M. xanthus* cells, which was readily distinguished from the A motility of wild-type cells by their relative differences in velocities [22]. Interestingly, the EPS^−^ cells, including strain SW2019 (A::Tn5, *ΔdifA*) and SW2021 (*ΔaglZ*, *ΔdifA*), also showed a rapid motility phenotype in methylcellulose, while cells lacking TFP, like strains SW538 (A::Tn5, *ΔpilA*) and SW2022 (*ΔaglZ*, *ΔpilA*), did not.

**Table 1:** Analysis of S motility.

Strains	Phenotypes			S motility On 0.3% MOPS Agar		S motility on Polystyrene in 1% Methylcellulose MOPS Medium	
	Motility*	Pili†	EPS‡	Isolated Cell§	Cell in Groups§	Isolated Cell§	Cell in Groups§
DK1622 (Wt)	A^+^ S^+^	+	+	–	+	+	+
MXH1216 (A::Tn5)	A^−^ S^+^	+	+	–	+	+	+
MXH2265 (*ΔaglZ*)	A^−^ S^+^	+	+	–	+	+	+
SW538 (A::Tn5, *ΔpilA*)	A^−^ S^−^	–	+	–	–	–	–
SW2022 (*ΔaglZ, ΔpilA*)	A^−^ S^−^	–	+	–	–	–	–
SW2019 (A::Tn5, *ΔdifA*)	A^−^ S^−^	+	–	–	–	+	+
SW2021 (*ΔaglZ, ΔdifA*)	A^−^ S^−^	+	–	–	–	+	+

*The motility phenotypes were initially determined on agar surfaces.

†The surface pili of the cells were confirmed by Western immunoblotting assays [48].

‡The EPS of strains were confirmed by the trypan blue binding assay [55].

§+, > 50% of the cells were motile; –, <5% of the cells were motile.

**Figure 1:**
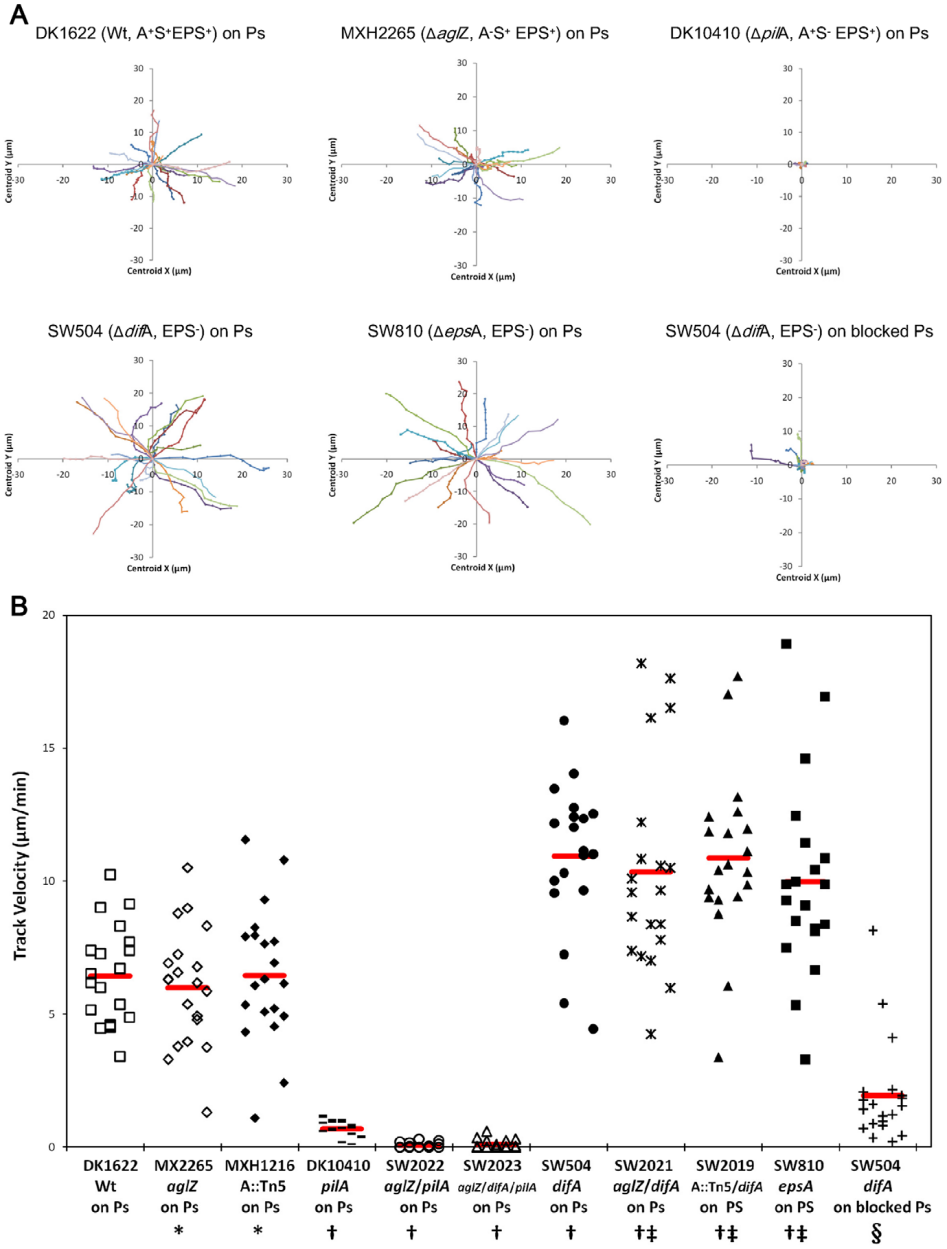
Tracking motility of *M. xanthus* isolated cells in 1% methylcellulose. Wild-type or different mutant cells were placed on different surfaces in 1% methylcellulose and cell movements were recorded by time lapse photography. Motility and tracks of 20 isolated cells were analyzed. Data are presented as tracking plots (panel A) and as diagrams (panel B) for the motile cells. ‘Ps’ represents polystyrene surface, and ‘blocked PS’ represents SuperBlock coated polystyrene surfaces. For strains DK1622, MXH1216, MXH2265, SW504, SW810, SW2019 and SW2021 on PS surfaces, only rapidly motile (velocity >1 µm/min) cells were counted. *, Student's *T*-test showed not statistically difference from DK1622 on Ps; †, Student's *T*-test *P* value<0.01 compared to DK1622 on Ps; ‡, Student's *T*-test showed no statistically difference from SW504 on Ps; §, Student's *T*-test *P* value<0.01 compared to SW504 on Ps.

To further study this phenomenon, different cells carrying mutations affecting A or S motility were placed on polystyrene surfaces in 1% methylcellulose-containing medium. The velocities of EPS^−^ cells, including strains SW504 (*ΔdifA*, Video S1) and SW810 (*ΔepsA*), were generally higher than the speed of wild-type cells (Fig. 1B). The movements of EPS^−^ cells in this system were not due to A motility, since strains SW2019 (A::Tn5, *ΔdifA*) and SW2021 (*ΔaglZ*, *ΔdifA*), derivatives of SW504 (*ΔdifA*) lacking A motility exhibited motility similar to their *ΔdifA* parent (Fig. 1). These rapid movements were characteristic of TPF-dependent motility. In contrast, in the respective mutant strains defective in surface pilus biogenesis, *ie.* DK10410 (*ΔpilA*), SW2022 (*ΔaglZ*, *ΔpilA*) and SW2023 (*ΔaglZ*, *ΔdifA*, *ΔpilA*), rapid motility was totally eliminated (Fig. 1A and B). Although EPS has been identified as the trigger for TFP retraction [23] and is considered a key component for S motility on agar [15], [16], [17], *M. xanthus* S motility on polystyrene surfaces in methylcellulose appeared to be independent of EPS.

### S motility of EPS^−^ *M. xanthus* cells in methylcellulose was dependent on the interaction of TFP with the polystyrene surface

**Figure 2:**
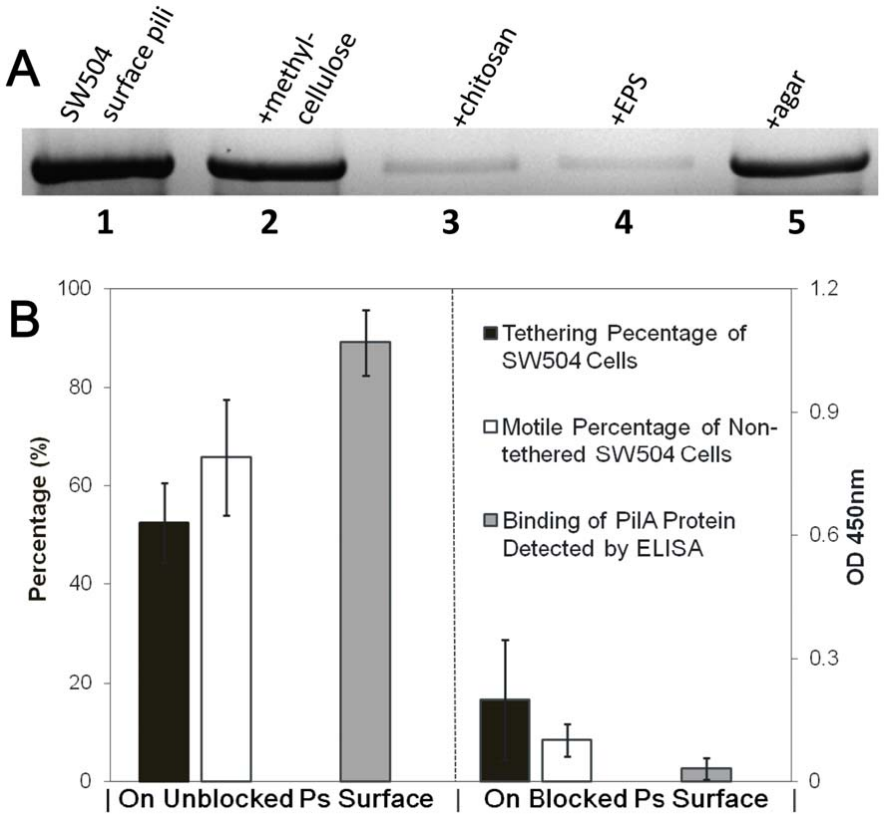
Effects of carbohydrates on TFP retraction, and the effects of blocking on SW504 (*ΔdifA*) cell movements and pilin binding. (A) Effects of carbohydrate on TFP retraction. Surface pilin from 5×10^8^ SW504 cells before (lane 1) or after incubating SW504 with methylcellulose (lane 2), insoluble chitosan suspension (lane 3), EPS suspension (lane 4) and granular agar suspension (lane 5) as described in *Materials and Methods*. PilA was probed with Western blotting. (B) The percentage of tethered vs. total SW504 cells (black columns) and ratio of motile cells (*v*>1 µm/min) in the non-tethered cells (white columns) were calculated from 20 random frames taken of cells on polystyrene surfaces *w/wo* blocking pretreatment. The amounts of PilA protein bound on these two surfaces were measured by ELISA, and shown are representative data for triplicate experiments. Means ± SD are plotted in panel B.

The retraction of TFP in EPS deficient mutants, such as strain SW504 (*ΔdifA*), is stimulated by mixing the cells with isolated EPS, which suggests the specific recognition of *M. xanthus* EPS by its TFP [23]. Using the retraction assay [23], the interactions of TFP with methylcellulose and agar were investigated. Methylcellulose was unable to trigger pilus retraction in EPS^−^ SW504 cells (lane 2, Fig. 2A), while insoluble chitosan (partially deacetylated chitin) and purified EPS were able to do so (lane 3 and 4, Fig. 2A), which excluded the possibility that methylcellulose replaced the role of EPS in *M. xanthus* S motility. Consistent with the S motility deficient phenotype of SW504 on agar, granular agar was also unable trigger the TFP retraction of SW504 cells (Lane 5, Fig. 2A).

Next, an ELISA assay was employed to evaluate the possible interaction between pilin and polystyrene surface. The purified PilA protein was shown to adhere onto the polystyrene surface and the protein's binding was remarkably reduced after coating the surface with SuperBlock buffer (Fig. 2B, grey columns). Consistent with the observed lack of PilA binding, the S motility of SW504 cells on the blocked surface was greatly inhibited (Fig. 1, Video S2) and their tethering abilities, motile percentages and velocities decreased significantly (Fig. 1 and Fig. 2B). These results suggested that the interaction between TFP and the polystyrene surface enable EPS^−^ *M. xanthus* cells to move by S motility in methylcellulose.

**Figure 3:**
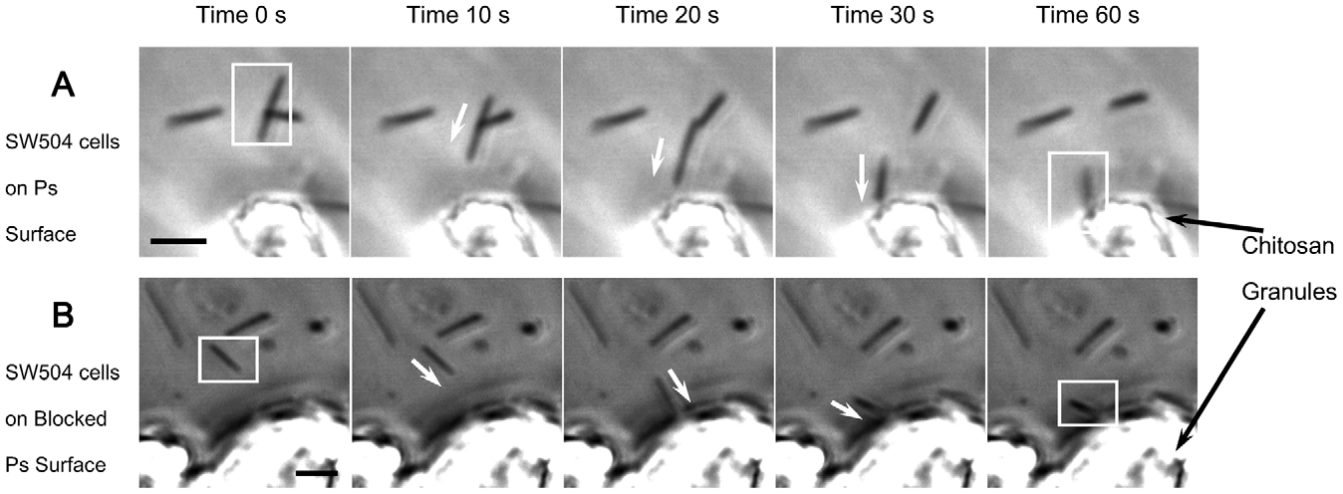
Chitosan triggered the S motility of SW504 (*ΔdifA*) on blocked surfaces in methylcellulose medium. These images were taken at 10 s intervals and placed in sequence. (A) shows SW504 cells on a unblocked polystyrene surface with a chitosan granule and (B) shows SW504 cells on a blocked polystyrene surface with a chitosan granule. White arrows indicate the direction of cellular movement and bars represent 5 µm.

To dispel the concern that the SuperBlock treatment of the surface might interfere with TFP retraction, a chitosan suspension, which is known to be able to trigger the retraction of *M. xanthus* TFP (Fig. 2A and [23]), was mixed with SW504 (*ΔdifA*) cells that were placed on the surfaces before adding the methylcellulose overlay. As shown in the serial images in Fig. 3, on both unblocked and blocked surfaces, SW504 cells located within a certain distance to chitosan granules (corresponding to the length of TFP) performed jerky motions towards chitosan granules (Video S3 and S4), which were most likely caused by the retraction of TFP. At the same time, similar motions were not observed when mixing SW504 cells with cellulose granules. These controls confirmed that the blocking treatment prevented binding of TFP on polystyrene surfaces but did not interfere with the general ability of TFP to retract.

### S motility of EPS^+^ *M. xanthus* cells on blocked polystyrene surfaces in methylcellulose

**Figure 4:**
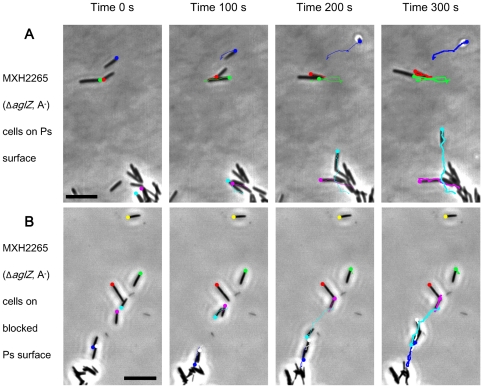
S motility of EPS^+^ *M. xanthus* MXH2265 (*ΔaglZ*) cells on unblocked and blocked polystyrene surfaces in methylcellulose. Serial images were taken and placed in sequence at 100 s intervals; bars represent 10 µm. Representative trajectories of isolated and group cell motilities are labeled with colorized lines and shown in images.

Further experiments were conducted to investigate the role of the interaction between TFP and the polystyrene surface in S motility of EPS^+^ cells. Such interactions were likely due to nonspecific binding compared to the recognition of *M. xanthus* EPS by TPF [23]. The blocking of the polystyrene surfaces had complicated influences on S motility of MXH2265 (*ΔaglZ*, A^−^EPS^+^) cells. On the blocked polystyrene surface at 1 hr, the S motility of isolated MXH2265 cells was inhibited and the motile percentage of cells was reduced to 33.2±8.3% from 81.5±5.2% on the unblocked polystyrene surface. On blocked surfaces, the motile percentage of MXH2265 (*ΔaglZ*, A^−^EPS^+^) isolated cells increased to 56.7±12.1% after 6 hrs incubation (Video S5), whereas the motile percentage of SW2021 (*ΔaglZ*, *ΔdifA,* A^−^EPS^−^) cells at 6 hr (13.5±5.3%) remained similar to the ratio observed at 1 hr (9.6±4.7%). By tracking the motility trajectories (Fig. 4, Video S6 and S7), on the unblocked surface, MXH2265 cells were observed to move and separate themselves from the cell groups (like the light blue trajectory shown in Fig. 4A). On the blocked surface, motility of isolated MXH2265 cells was inhibited at 1 hr, while the cells in groups remained motile (Fig. 4B). These results suggested that S motility of the isolated EPS^+^ cells was partially dependent on the nonspecific interaction of TFP with polystyrene surfaces in methylcellulose.

### EPS^+^ *M. xanthus* cells deposit EPS on both blocked and unblocked surfaces to enable surface-independent movement

**Figure 5:**
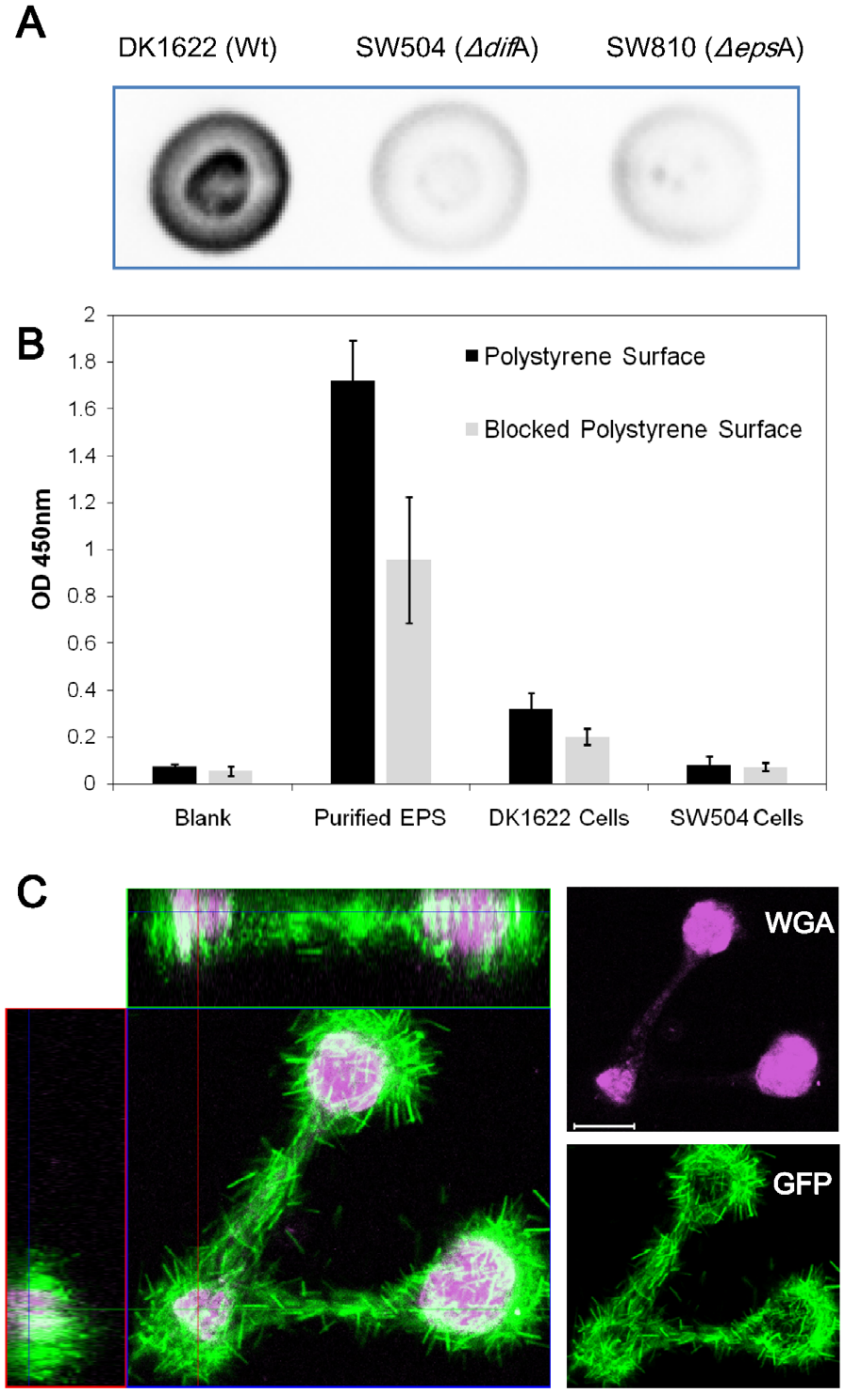
Detection of EPS on polystyrene and agar surfaces. (A) Dot blots probed with anti-EPS antiserum. Cell lysate from 5×10^7^ DK1622, SW504 (*ΔdifA*) or SW810 (*ΔepsA*) cells were applied to each spot, respectively. (B) Purified EPS, DK1622 cells or SW504 cells were placed on the unblocked/blocked polystyrene surface, respectively, and the adhered EPS was detected by ELISA utilizing anti-EPS antiserum. The experiments were done in triplicate; means ± SD of the absorbance at 450 nm are plotted. (C) Orthogonal sections of the DK10547 12 hr swarm on agar surfaces. The distribution of cells was revealed by GFP (green) and EPS was detected with Alexa 633-WGA (pink). Images were obtained with a 63× objective using CLSM as described; bar = 20 um.

We noticed that about 30% of isolated EPS^+^ cells were still motile on the blocked surface, while only about 9% of EPS^−^ cells were able to move under the same condition (Fig. 2B). One possible explanation for this difference is that EPS^+^ cells would eventually deposit sufficient EPS on the blocked surface to support single-cell S motility. In order to detect the EPS attached on the surface, a polyclonal anti-EPS antiserum was prepared using purified wild-type EPS. Dot blots showed that this antibody was able to recognize the EPS of wild-type cells, and had a low background for EPS^−^ cells (Fig. 5A). Consistent with our hypothesis, purified EPS was shown to bind to the polystyrene surface even after treatment with blocking buffer (Fig. 5B). Considerable amounts of EPS were detected on both blocked and unblocked surfaces, after washing off the wild-type cells, while neither surface retained any detectable amounts of EPS after exposure to SW504 (*ΔdifA*) cells (Fig. 5B). Since the ELISA used to detect EPS on polystyrene surfaces could not be applied to agar surfaces, confocal laser scanning microscope (CLSM) with specific lectin staining was employed to reveal the EPS shed on agar surfaces. The results showed that DK10547 cells (Fig. 5C-green), a *gfp*-expressing derivative of DK1622 [31], followed the EPS trails (Fig. 5C-pink) and aggregated on spots with high EPS concentration, which also suggested the presence of an agar surface covered by EPS under *M. xanthus* swarms.

### S motility of EPS^−^ *M. xanthus* cells in groups is not coordinated on polystyrene surface in methylcellulose

**Figure 6:**
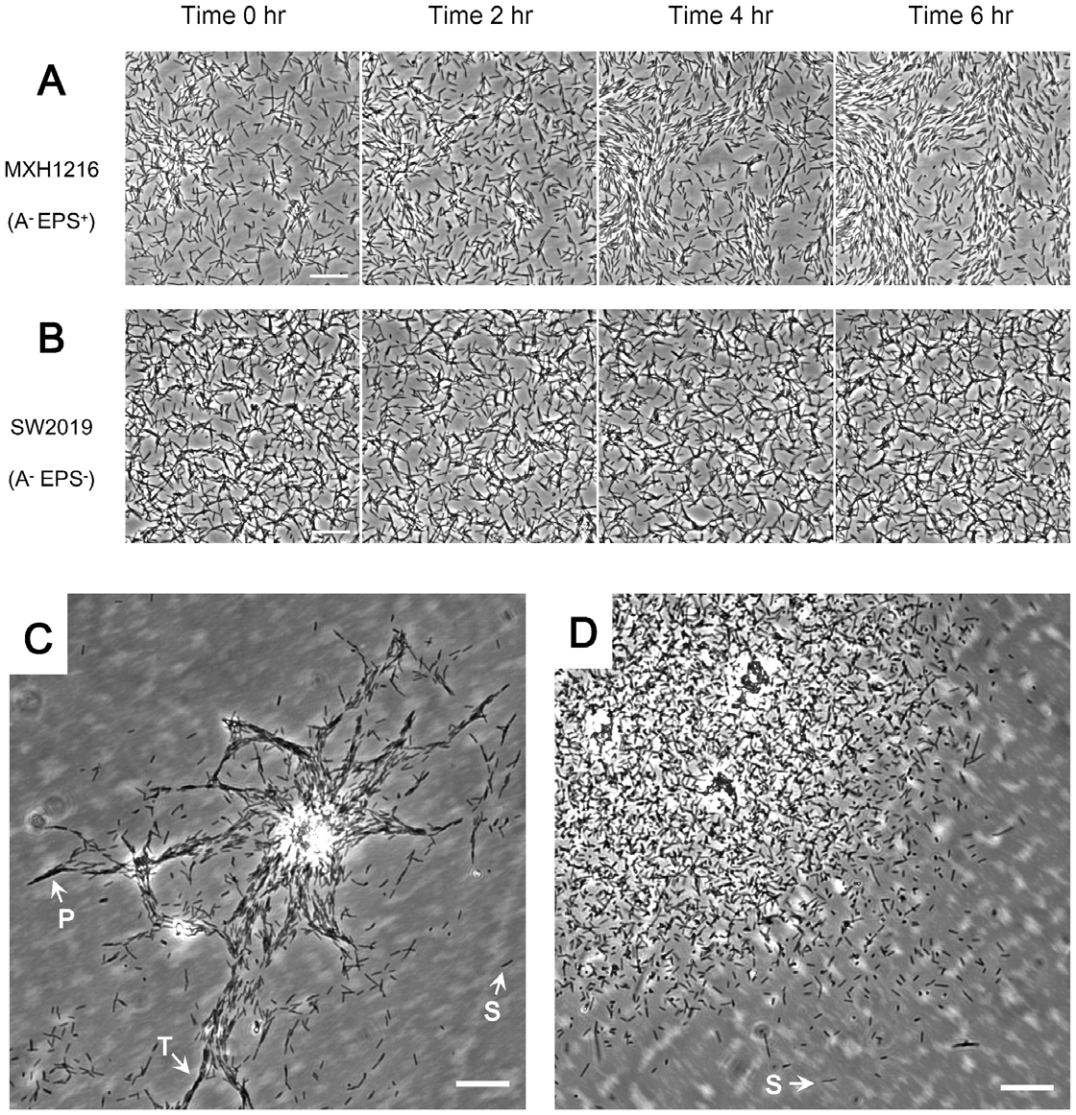
Movements of EPS^+^ and EPS^−^ cells in groups on polystyrene surface in methylcellulose medium. Serial images were taken of high cell density spots of strains MXH1216 (A::Tn5, in panel A) and SW2019 (A::Tn5, *ΔdifA*, in panel B), and placed in sequence at 2 hr intervals; bars represent 20 µm. Movements of strain MXH1216 (C) and SW2019 (D) in spreading zones; bars represent 30 µm. Isolated single cells (S), peninsulas (P) and trails (T) structures are labeled in panels C and D.

The S motility of EPS^+^ cells in groups is most likely dependent on EPS regardless of the surfaces involved and EPS has been suggested to be important for coordinated movements in *M. xanthus* [32], [33]. Our results showed that the S motility of EPS^−^ *M. xanthus* cells in groups was not coordinated on polystyrene surface in methylcellulose (Fig. 6). With the EPS and cell-cell contact, EPS^+^ cells in groups formed initial multi-cellular aggregates after being incubated in methylcellulose at 32°C for 6 hr, and the cellular movement was apparently coordinated (Fig. 6A). However, the EPS^−^ cells only exhibited random movements in groups and did not form aggregates even after 6 hr incubation in methylcellulose (Fig. 6B). By examining the cellular distribution in the spreading zones, some specific swarming structures, like peninsulas and trails (Fig. 6C), were indentified in EPS^+^ cells, and such structures are usually observed in the swarms resulting from S motility of *M. xanthus* on agar [16], [24]. However, the spreading zones of EPS^−^ cells were most composed of randomly distributed single cells (Fig. 6D). These results confirmed that, in methylcellulose, EPS was likely a key component in coordinating movements in groups of cells.

## Discussion

The force-generating mechanism involved in *M. xanthus* S motility was shown to involve the extension and retraction of pili [21], [22], [34], which is triggered by the specific interaction between TFP and EPS [23]. The requirement for EPS in *M. xanthus* S motility on agar surfaces has been previously demonstrated [15], [16], [17]. However, in the present study we found that in methylcellulose, nonspecific interactions of TFP with the polystyrene surface compensated for the absence of EPS as an anchoring substratum and allowed single-cell TFP-dependent motillity in *M. xanthus*. Consistent with our findings, the TFP of different bacteria can bind to a variety of surfaces, including inert surfaces, bacterial cells, and eukaryotic cells, and can mediate both colonization of the surfaces and intimate contact through pili retraction [7], [35]. Similar to S motility in *M. xanthus*, the twitching motility in *P. aeruginosa* and *N. gonorrhoeae* is primarily a social activity. However, it has also been shown that isolated cells move by twitching motility when in contact with different surfaces [28], [29]. The nonspecific interaction of TFP with the surface might also play an important role in the aforementioned twitching motility of isolated cells.

We also observed that the presence of a general anchoring surface in a viscous environment could not substitute for the role of cell surface EPS in cell group movements of *M. xanthus*. Coordinated movements are required for the swarming and aggregation of A^−^S^+^ cell groups on agar surfaces [24]. On polystyrene surfaces in methylcellulose, the TFP-dependent motility of EPS^−^ cells was not coordinated and cells failed to aggregate or perform group swarming. For EPS^+^ cells, TFP specifically recognized and bound onto the EPS of an adjacent cell or in the slime trail [23], which coordinated cellular group formation into peninsulas, trails and swarm aggregates. The asymmetry of EPS distribution might regulate the movements in *M. xanthus* swarming, while the uniformity of nonspecific anchoring surface distribution might not do so. Our study provided the direct evidence for a definitive role for EPS in coordinating S motility in cellular groups, which is another important biological function of EPS in *M. xanthus*.

**Figure 7:**
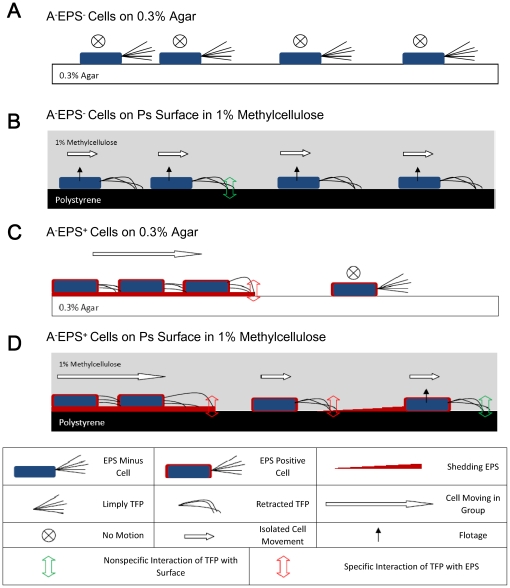
Model for S motility of *M. xanthus* isolated cells in methylcellulose medium. (A) A^−^EPS^−^ *M. xanthus* cells on 0.3% agar surfaces. The absence of EPS results in defects in S motility. (B) A^−^EPS^−^ *M. xanthus* cells on polystyrene surface in 1% methylcellulose medium. The nonspecific interaction of TFP with surface activates the S motility of isolated EPS^−^ cells. (C) A^−^EPS^+^ *M. xanthus* cells on 0.3% agar surface. The interaction between TFP and EPS present in the shed extracellular matrix and on the cell surface triggers the S motility of cells in groups, while isolated cells fail to move by S motility because of the absence of adjacent EPS material. (D) A^−^EPS^+^ *M. xanthus* cells on polystyrene surface in 1% methylcellulose medium. Isolated cells move by nonspecific interaction of TFP with surfaces, and shed EPS from isolated motile cells or cells in groups also triggers single-cell S motility. Cells in groups move by normal S motility. Panels A and C are adapted from the model previously proposed [23].

Our results for S motility of *M. xanthus* isolated cells in methylcellulose medium are summarized in Fig. 7. A^−^EPS^−^ *M. xanthus* cells were deficient in S motility on agar because of the absence of EPS to bind with TFP and trigger retraction (Fig. 7A, adapted from [23]). Inert surfaces, like polystyrene and glass, are normally employed in the methylcellulose motility assays [22], [30]. On these surfaces, the A^−^EPS^−^ cells display uncoordinated S motility. It has been observed in previous studies that polystyrene surfaces allowed tethering of TFP [22] and the BSA-coated polystyrene surface reduced the amount of tethering by *M. xanthus* wild-type cells [36]. Consistent with these results, our findings suggest that polystyrene provided an anchoring surface for S motility of EPS^−^ cells in methylcellulose (Fig. 7B). Results similar to those obtained with the cells on the polystyrene surface were obtained with cells on a glass surface (data not shown), which indicated that TFP also interacted nonspecifically with glass.

S motility of isolated *M. xanthus* EPS^+^ cells has been observed on polystyrene surface in methylcellulose [22] but rarely on 1.5% agar [9]. The need for adjacent EPS might explain why *M. xanthus* cells normally move by S motility in groups (Fig. 7C, adapted from [23]). In our model (Fig. 7D), single-EPS^+^ cell S motility in methylcellulose system can be divided into two categories: one involves activation by the nonspecific interaction of TFP with the polystyrene surface and the other is triggered by the binding of TFP to the EPS deposited on the surfaces. Motile individual cells initially attached to virgin surfaces could modify the surface by shedding their EPS during movement, and subsequently provide a specific anchor for other isolated cells to display S motility. Consistent with this assumption, it was observed that inhibition of the S motility of isolated EPS^+^ cells could be overcome following incubation, which possibly may result from the development of a blocked surface due to the shedding of EPS from the cells.

In addition, single-cell S motility of *M. xanthus* was only observed under certain conditions invovling high water content (e.g. 1% methylcellulose solution), which might have a bio-ecological relevance for *M. xanthus*. Cellular motility provides bacteria with the capacity to actively seek out favorable environments and avoid hazardous situations, thereby facilitating growth and survival in nature [3]. Myxococci have been commonly found in terrestrial habitats [37], [38] and they are also suggested to be adapted to environments with periodic dry spells as well as living permanently in fresh water habitats [39]. At the same time, some *Myxococcus* strains have been isolated from aqueous samples [40], which indicates that these bacteria might be even more widely spread. In the environments with high water content, *M. xanthus* cells mainly depend on their S motility to travel and colonize [13]. Thus, the EPS independent single-cell S motility described in this report could possibly promote rapid exploratory movement of colonies over newly hydrated surfaces.

However, the role of cell floating in this S motility is still unknown. In the methylcellulose motility assay, the 1% methylcellulose most likely provides a highly viscous medium rather than replacing the function of EPS. In methylcellulose, the Brownian motion of the cells is greatly reduced and gliding motility could be readily examined [22]. The buoyancy provided by the liquid medium could reduce the cell body weight acting on the surface and subsequently the resistance to motility caused by friction between a cell body and the surface. Under these conditions, S motile cells might not need to bind as tightly to the EPS and the nonspecific binding may be sufficient to activate S motility.

## Materials and Methods

### Bacterial growth conditions and construction of strains

*M. xanthus* cells were grown in CYE medium [41] at 32°C on a rotary shaker at 300 rpm for 24 hr and harvested by 5,000×g centrifugation for 10 min. The *M. xanthus* strains used in this study are listed in Table 2. Mx4-mediated generalized transduction [41] was used to construct strains SW2019 (A::Tn5-*lac*Ω1215, *ΔdifA*) and SW2020 (A::Tn5-*lac*Ω1215, *ΔdifA*, *ΔpilA*). SW2019 and SW2020 were constructed by transducing an Mx4 lysate of MXH1216 (A::Tn5-*lac*Ω1215) into SW504 (*ΔdifA*) and SW2017 (*ΔdifA*, *ΔpilA*), respectively, and selecting for kanamycin (Km) resistance. An in-frame deletion plasmid of *aglZ* was generated using MXH2265 (*ΔaglZ*) chromosomal DNA as the PCR template with the primers AGLZ-up (CCGGAATTCAACCGCCCGATAGGATGTTC) and AGLZ-down (CGCGGATCCTGGCGTACTCCCACTCGTAGAG). The amplified regions were digested with EcoRI and BamHI and ligated into similarly digested pBJ113 [42] to create the plasmids pBJ113-aglZ. After being verified by sequencing, the deletion plasmid pBJ113-aglZ was electroporated into SW504 (*ΔdifA*), DK10410 (*ΔpilA*) and SW2017 (*ΔdifA, ΔpilA*) to construct SW2021 (*ΔaglZ*, *ΔdifA*), SW2022 (*ΔaglZ*, *ΔpilA*) and SW2023 (*ΔaglZ*, *ΔdifA, ΔpilA*), respectively. Deletion mutants were selected by their galactose resistant and kanamycin sensitive phenotypes and confirmed with PCR and sequence analyses.

**Table 2:** *M*. *xanthus* strains used in this study.

Strains	Relevant genotype	Relevant phenotype*	Ref. or Source
DK1622	Wild type (Wt)	A^+^ S^+^ Pilus^+^ EPS^+^	[51]
DK10410	DK1622, *ΔpilA*	A^+^ S^−^ Pilus^−^ EPS^+^	[48]
DK10547	*gfp*-expressing derivative of Wt	A^+^ S^+^ Pilus^+^ EPS^+^	[31]
MXH1216	DK1622, A::Tn5-*lac*Ω1215†	A^−^ S^+^ Pilus^+^ EPS^+^	[52]
MXH2265	DK1622, *ΔaglZ*	A^−^ S^+^ Pilus^+^ EPS^+^	[53]
SW504	DK1622, *ΔdifA*	A^+^ S^−^ Pilus^+^ EPS^−^	[17]
SW538	DK1622, A::Tn5-*lac*Ω1215, *ΔpilA*::*Tc* ^r^	A^−^ S^−^ Pilus^−^ EPS^+^	[32]
SW810	DK1622, *ΔepsA*	A^+^ S^−^ Pilus^+^ EPS^−^	[15]
SW2017	DK1622, *ΔdifA*, *ΔpilA*	A^+^ S^−^ Pilus^−^ EPS^−^	[54]
SW2019	DK1622, A::Tn5-*lac*Ω1215, *ΔdifA*	A^−^ S^−^ Pilus^+^ EPS^−^	This study
SW2020	DK1622, A::Tn5-*lac*Ω1215, *ΔdifA*, *ΔpilA*	A^−^ S^−^ Pilus^−^ EPS^−^	This study
SW2021	DK1622, *ΔaglZ, ΔdifA*	A^−^ S^−^ Pilus^+^ EPS^−^	This study
SW2022	DK1622, *ΔaglZ, ΔpilA*	A^−^ S^−^ Pilus^−^ EPS^+^	This study
SW2023	DK1622, *ΔaglZ*, *ΔdifA*, *ΔpilA*	A^−^ S^−^ Pilus^−^ EPS^−^	This study

*‘A’ represents A motility, and ‘S’ represents S motility. S motility phenotype of these strains was determined on agar surfaces.

†The strain was constructed by MacNeil *et al*. with a Tn5-lac insertion into a gene cluster for A motility [52].

### 0.3% agar assay for S motility

Swarm plates (MOPS medium solidified with 0.3% Bacto agar) were inoculated and S motility on agar surfaces was recorded as described previously [13]. Plates were incubated at 32°C for 1 hr before imaging. The movements of individual cells in groups were tracked employing the tetrazolium staining assay [43].

### Methylcellulose assay for S motility

This assay was based on a previously published protocol [22] with the following modifications. The harvested *M. xanthus* cells were diluted in MOPS buffer (10 mM MOPS, 8 mM MgSO_4_, pH 7.6) to 5×10^6^ cell/ml. One microliter of cells was transferred onto different surfaces (described below) and allowed to settle for 10 min. The cells were then carefully overlaid with 200 µl of 1% methylcellulose in MOPS buffer and placed at 32°C for 1 hr before recording. For the methylcellulose assay with insoluble polysaccharides, 5 µl of a 1 mg/ml chitosan suspension (molecular weights from 100K to 300K, Acros Organics, USA) or cellulose suspension (Acros Organics) was added to the cells prior to the methylcellulose overlay.

The motility of *M. xanthus* cells in 1% methylcellulose was tested on different surfaces. Polystyrene plates (Costar™ cell culture plates, Fisher Scientific, USA) were used as a polystyrene surface material (called Ps surface in this paper). The SuperBlock pre-treated polystyrene surface (called blocked-Ps surface in this paper) was prepared by coating 24-well polystyrene plates with 600 µl SuperBlock™ T20 (PBS) blocking buffer (Thermo Scientific, USA) three times according to the manufacturer's instructions.

### Microscopic imaging and analysis of motility in methylcellulose

Cell movements were monitored with a Nikon Eclipse TE200 inverted microscope through a 60× objective, captured with a SPOT RT740 CCD camera (Diagnostic Instrument, USA) and recorded with SPOT advanced software (Version 4.6, Diagnostic Instrument). A heat plate system (Brook Instrument Corp, USA) was mounted onto the stage of the microscope to maintain the culture temperature at 32°C. Continuous images were taken at 10 s intervals and stored as TIFF image sequence files. For all the Supporting Information Videos, Microsoft videos (5 frames/s, wmv file) were exported with SPOT software resulting in a 50× faster than real-time replay speed.

For velocity measurements and trajectory tracking, the TIFF image sequences were imported to Manual Tracking [44], a plug-in for ImageJ software [45]. For each condition, 20 different cells moving along their long axes were tracked at their leading poles for 2 min (13 frames). Velocity was calculated with the Manual Tracking plug-in by measuring the distance from a starting to an ending point within two frames. A static synthetic view of cell motility tracks was generated and the recorded xy coordinates were exported to Microsoft Excel software to present the data as plots. One color was applied for each trajectory.

To calculate the percentage of tethered cells (with one end of the cell attached to the solid surface such that the cell body was lifted up), 20 random frames were selected and the tethered cells were manually counted as previously described [36]. Motile percentages were calculated by estimating the number of motile versus total non-tethered cells as previously described [46]. Cells were defined as isolated single cells if the individual cells were separated from other cells by a distance greater than approximately one cell length [24].

### TFP retraction assay

The effects of different complex carbohydrates on TFP retraction were evaluated with a previously described mixing assay [23]. The purified EPS was isolated from *M. xanthus* DK1622 and quantified as previously described [23], [47]. To test the rescue of hyperpiliated EPS^−^ mutants by different complex carbohydrates, about 5×10^8^ EPS^−^ SW504 cells were mixed with purified EPS (1 mg/ml carbohydrate), insoluble chitosan (1 mg/ml, 100-300K), methylcellulose (1 mg/ml, Fisher) or granular agar (1 mg/ml, Fisher) prior to incubation in cohesion buffer at 32°C for 30 min [23]. Cell-surface pili were then analyzed with Western blotting using anti-pilA antibody [36] as previously described [48].

### Generation and purification of anti-EPS antiserum

Purified EPS from DK1622 cells [23], [47] was used to challenge two rabbits to raise polyclonal anti-EPS antibodies. Immunizations were performed by GenScript (USA) based on established protocols [49]. The antiserum was preabsorbed with acetone powder prepared from the EPS deficient mutant strain SW504 (*ΔdifA*) as described by Harlow & Lane [49].

### Immuno dot blots

Dot blots of whole-cell lysates were performed according to standard protocols [49]. Cell pellets of DK1622, SW504 and SW810 were collected and suspended in 1% SDS solution at 5×10^9^ cell/ml. Cells were then lysed by boiling for 10 min. Ten µl of each cell lysate was applied to a single spot on a nitrocellulose membrane and probed with the anti-EPS antiserum (1∶200 diluted).

### ELISA for Pilin and EPS adsorbed on different surfaces

Purified truncated pilin (PilA^(29–220)^) protein and anti-PilA antibody were prepared as previously described [36]. For ELISA, truncated PilA protein was dissolved in deionized water to a concentration of 10 µg/ml. Then 50 µl of PilA solution was added to the wells of Costar™ polystyrene 96-well plates *w/wo* SuperBlock pre-treatment, and 50 µl water was used as control. The plates were incubated at 37°C for 1 hr. Next, the PilA protein adsorbed on the surface of the well was detected with a standard ELISA [49]. The anti-PilA antibody used in this assay was 2000-fold diluted and peroxidase-conjugated goat anti-rabbit IgG was 5000-fold diluted. The optical density at 450 nm was determined and data was calculated as the average of five repeats. To detect the EPS shed from *M. xanthus* cells onto different surfaces, 50 µl DK1622 or SW504 cells at 1×10^8^ cell/ml in MOPS buffer was added to the wells of Costar™ polystyrene 96-well plates *w/wo* SuperBlock pre-treatment while 50 µl of MOPS buffer and 50 µl purified EPS (10 µg/ml carbohydrate in MOPS buffer) were used as controls. ELISA for EPS was performed as described above, except that the anti-EPS antiserum was 1000-fold diluted.

### EPS staining and confocal microscopy

MOPS agar layers in cover slide bottom chambers were prepared following previously described methods [50]. *M. xanthus* DK10547, a *gfp*-expressing derivative of DK1622 [31], was spotted on the agar surface and kept in a humidity chamber for a 12 hr incubation periods. Carbohydrates present in the EPS portion of the cell swarm were stained with 10 µg/ml of Alexa633-conjugated derivatives of wheat germ agglutinin lectin (WGA, Molecular Probes, USA) in MOPS buffer [50].

The specimens were viewed using a PASCAL 5 confocal laser scanning microscope (Zeiss, Jena, Germany) after a 30 min incubation period at 32°C in the dark. The scanning module of the system was mounted onto an inverted microscope (Axiovert 200 M). Excitation at 488 nm with an argon laser in combination with a 505–530 nm bandpass emission filter were used for imaging of GFP-expressing cells. 633 nm excitation with a helium-neon laser and a 650 nm longpass emission filter were used to reveal Alexa633-conjugated lectin. Images were acquired through a 63x/NA1.4 oil lens.

## Supporting Information

Video S1 *M. xanthus* SW504 (*ΔdifA*) cells were placed on unblocked polystyrene surface in MOPS medium containing 1% methylcellulose, and cell movements were recorded as described in *Materials and Methods*.
(WMV)

Video S2 *M. xanthus* SW504 (*ΔdifA*) cells were placed on blocked polystyrene surface in MOPS medium containing 1% methylcellulose, and cell movements were recorded.
(WMV)

Video S3 *M. xanthus* SW504 (*ΔdifA*) cells were mixed with insoluble chitosan suspension (1 mg/ml) and then placed on unblocked polystyrene surface in MOPS medium containing 1% methylcellulose, and cell movements were recorded.
(WMV)

Video S4 *M. xanthus* SW504 (*ΔdifA*) cells were mixed with insoluble chitosan suspension (1 mg/ml) and then placed on blocked polystyrene surface in MOPS medium containing 1% methylcellulose, and cell movements were recorded.
(WMV)

Video S5 *M. xanthus* MXH2265 (*ΔaglZ*) cells were placed on blocked polystyrene surface in MOPS medium containing 1% methylcellulose and incubated at 32°C for 6 hr, and cell movements were recorded.
(WMV)

Video S6 *M. xanthus* MXH2265 (*ΔaglZ*) cells were placed on unblocked blocked polystyrene surface in MOPS medium containing 1% methylcellulose, cell movements were recorded, and the motile trajectories of selected cells were tracked and recoded as described in *Materials and Methods*.
(WMV)

Video S7 *M. xanthus* MXH2265 (*ΔaglZ*) cells were placed on blocked polystyrene surface in MOPS medium containing 1% methylcellulose, cell movements were recorded, and the motile trajectories of selected cells were tracked and recoded.
(WMV)
